# Quantitative ultrasound approaches for diagnosis and monitoring hepatic steatosis in nonalcoholic fatty liver disease

**DOI:** 10.7150/thno.40249

**Published:** 2020-03-04

**Authors:** Amir M. Pirmoazen, Aman Khurana, Ahmed El Kaffas, Aya Kamaya

**Affiliations:** 1 Department of Radiology, School of Medicine, Stanford University, Stanford, California; 2 Department of Radiology, University of Kentucky, Lexington, Kentucky; 3 Department of Radiology, Molecular Imaging Program at Stanford, School of Medicine, Stanford University, Stanford, California

**Keywords:** nonalcoholic fatty liver disease, nonalcoholic steatohepatitis, quantitative ultrasound, noninvasive assessment, hepatic steatosis

## Abstract

Nonalcoholic fatty liver disease is a major global health concern with increasing prevalence, associated with obesity and metabolic syndrome. Recently, quantitative ultrasound-based imaging techniques have dramatically improved the ability of ultrasound to detect and quantify hepatic steatosis. These newer ultrasound techniques possess many inherent advantages similar to conventional ultrasound such as universal availability, real-time capability, and relatively low cost along with quantitative rather than a qualitative assessment of liver fat. In addition, quantitative ultrasound-based imaging techniques are less operator dependent than traditional ultrasound. Here we review several different emerging quantitative ultrasound-based approaches used for detection and quantification of hepatic steatosis in patients at risk for nonalcoholic fatty liver disease. We also briefly summarize other clinically available imaging modalities for evaluating hepatic steatosis such as MRI, CT, and serum analysis.

## Introduction

Nonalcoholic fatty liver disease (NAFLD) is considered to be the hepatic manifestation of metabolic syndrome and is dramatically increasing in prevalence, paralleling the global obesity epidemic 1,2. Nonalcoholic fatty liver disease is the most common subtype of hepatic steatosis and the most common chronic liver disease in the Western world. It refers to a broad spectrum of disease defined by excessive and abnormal accumulation of fat in hepatic cells in the absence of clinically significant alcohol intake, viral infection, or other etiologies that can lead to hepatic steatosis 3. The prevalence of NAFLD is estimated to be 30% in Western populations and up to 90% in patients with insulin resistance, obesity, dyslipidemia, hypertension, and genetic predispositions 4. A recent study in 43,166 patients in Korea showed that NAFLD was strongly associated with increased risk of type 2 diabetes mellitus in euglycemic patients 5. Nonalcoholic fatty liver disease ranges from nonalcoholic fatty liver (NAFL) to nonalcoholic steatohepatitis (NASH) based on histologic analysis. Nonalcoholic fatty liver includes early stages of hepatic steatosis with or without mild lobular inflammation, while the hallmark of NASH is coexisting hepatic inflammation. NASH is further subclassified based on varying degrees of fibrosis which progress to frank cirrhosis. Though NAFL and early-stage NASH may be reversible, hepatic cirrhosis is irreversible and may progress to decompensated liver cirrhosis 6. In fact, NASH is expected to become the most common etiology for liver transplantation in the next 10-20 years 7,8. Patients with NASH are at increased risk of liver-related death with an estimated rate of 2-5% every 10 years. This risk further increases with decompensated cirrhosis. Moreover, NASH is associated with higher risk of cardiovascular-related death 8,9.

Early detection of NAFLD, continuous monitoring, and early therapeutic intervention, such as lifestyle modifications as well as treatment with pharmaceutical agents (currently in clinical trials) can improve overall patient outcomes and potentially decrease the economic burden on healthcare costs. Other patient subgroups such as those undergoing chemotherapy or bariatric surgery are at risk of development of fatty liver and may also benefit from monitoring of hepatic fat fraction. Non-targeted liver biopsy remains the gold standard for diagnosis and grading of hepatic steatosis, fibrosis, and inflammation 10. Hepatic steatosis is diagnosed when more than 5% of steatotic hepatocytes are microscopically seen in a liver tissue section. This threshold has provided a reference standard for evaluating non-invasive quantitative methods. However, the limitations of biopsy include that biopsy is an invasive procedure with potential risks of pain, bleeding, infection, and rarely, death 11. Other limitations of liver biopsy include the subjective nature of pathologic assessment of hepatic steatosis, the ordinal rather than continuous scale of assessment, and the fact that histologic assessment relies on manual counting of the number of affected hepatocytes, rather than quantifying the relative volume of lipid within a sample. Further, liver biopsy is subject to sampling variabilities due to small tissue sample; therefore, alternative noninvasive biomarkers for screening and monitoring of patients with hepatic steatosis are highly desirable.

Here, we summarize methods in grading of NAFLD, including non-imaging biomarkers and corresponding scoring systems currently in use for managing hepatic steatosis. We then provide a comprehensive review of imaging in liver fat quantification, focusing on innovative QUS approaches studied in human subjects (Table 1). Finally, we provide a brief overview of alternative non-ultrasound based imaging techniques, which are clinically available for detection and characterization of hepatic steatosis, particularly in NAFLD.

## Non-imaging biomarkers for evaluation of liver fat

Several non-imaging biomarkers such as electrical impedance tomography 12 along with steatosis scoring systems, including SteatoTest ™ (Biopredictive, Paris, France); Fatty Liver Index; Hepatic Steatosis Index; Lipid Accumulation Product; Index of NASH; and NAFLD Liver Fat Score 13 have been studied as alternative noninvasive methods of evaluating liver fat. These are obtained from blood biomarkers such as alanine transaminase (ALT); a2-macroglobulin; apolipoprotein A-1; haptoglobin; total bilirubin; gamma-glutamyl transferase; total cholesterol; triglycerides; glucose; and various risk factors such as age, gender, and body mass index (BMI). Among these scoring systems, only SteatoTest has been proven to hold high diagnostic accuracy when validated against liver biopsy 14; others have only been validated against conventional qualitative ultrasound, which is a poor indicator of hepatic steatosis 13. In general, chemical biomarkers and scoring systems derived from them have all shown relatively low sensitivity for diagnosis and monitoring of NAFLD. For example, persistent elevation of ALT can indicate fibrosis and disease progression; ironically, patients may still have normal liver enzymes during advanced stages of NAFLD. Indeed, elevated ALT has poor sensitivity and specificity in detecting NASH (45% and 85%, respectively) 15. Further, NAFL and NASH are more prevalent (76% and 56%, respectively) in patients with type 2 diabetes mellitus despite normal ALT levels 16. To date, the best-validated biomarker for predicting the severity of NAFLD is the NAFLD fibrosis score (NFS) 17. This score is calculated by accounting various risk factors: age; BMI; hyperglycemia; platelet count; albumin; and AST-to-ALT ratio. However, NFS has only 75% sensitivity and 58% specificity in discerning advanced liver fibrosis. Newer studies have investigated the utility of Cytokeratin 18 (a byproduct of caspase 3 mediated hepatocyte injury) 18 and more recently, microRNAs as potential chemical biomarkers; however, these have shown relatively poor sensitivity and specificity in detecting NASH 19,20, and further studies are needed to validate their diagnostic performance 21.

## Conventional ultrasound

**Figure 1:**
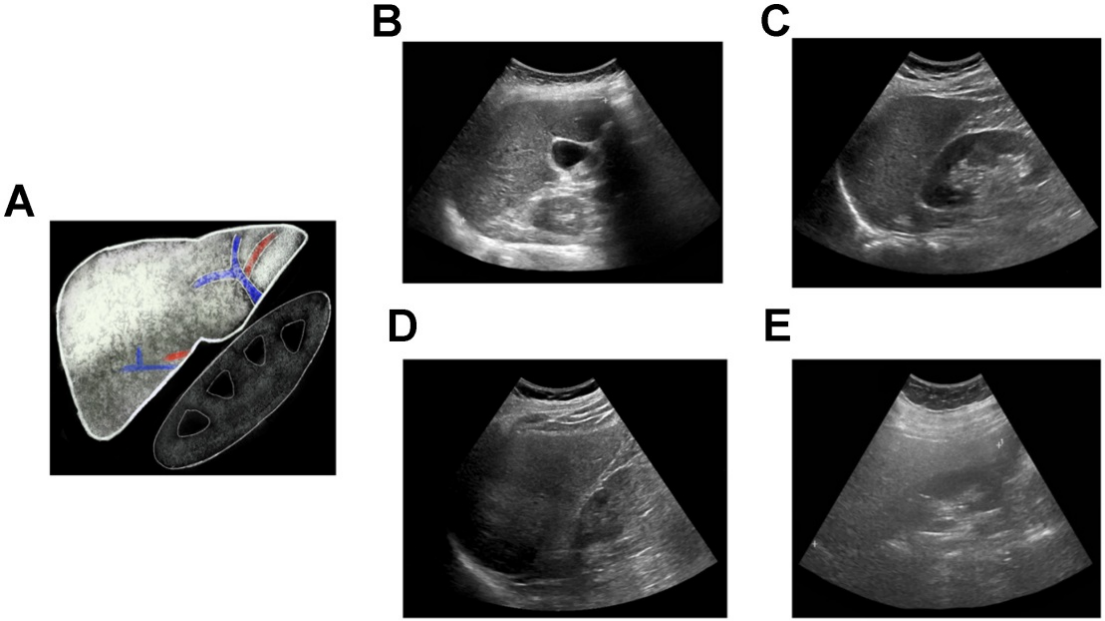
Qualitative assessment of liver fat with conventional ultrasound. **(A)** Schematic showing classic qualitative features of fatty liver - increased echogenicity compared to right kidney, blurring of intrahepatic vessels and posterior beam attenuation. Clinical ultrasound images demonstrating **(B)** normal, **(C)** mild, **(D)** moderate, and **(E)** severe fatty liver.

Conventional ultrasound (i.e., grayscale abdominal ultrasound evaluation of the liver) is the most common imaging modality for subjective evaluation of hepatic steatosis, with good sensitivity and specificity in detecting moderate to severe levels of steatosis (84.8% and 93.6%, respectively) 22; however, overall sensitivity and specificity has been reported to be moderate (65% and 81%, respectively) 23 due to variability in discerning mild hepatic steatosis. Hepatic steatosis is typically classified using B-mode images as none, mild, moderate, or severe (Figure 1). This assessment is based on observations including 1) increased liver echogenicity compared to the renal cortex; 2) decreased conspicuity of hepatic vasculature; 3) presence of focal fat sparing; and 4) decreased ability to visualize the diaphragm and deeper liver parenchyma. The last observation is caused by ultrasound beam attenuation from interfaces introduced by intra-hepatocyte fatty accumulation 24. Ultrasound has multiple advantages compared to other imaging modalities, such as ease of use, portability, accessibility, real-time capability, and relatively low cost. However, simple grayscale ultrasound has relatively poor interobserver agreement due to its subjective nature 25, along with reduced sensitivity in detecting mild hepatic steatosis. In addition, there is some overlap between the appearance of steatosis and fibrosis if only B-mode images are qualitatively assessed 26. To address these limitations, more recent studies have focused on QUS approaches for characterizing and classifying hepatic steatosis (Table 1) that will be discussed in more detail below.

## Quantitative ultrasound-based techniques

Several QUS techniques have been studied to improve the diagnosis and classification of hepatic steatosis in NAFLD. These include controlled attenuation parameter (CAP) measured by transient elastography (TE) device; attenuation (AC) and backscatter coefficients (BSC); computerized calculation of hepatorenal index (HRI); and ultrasound envelope statistic parametric imaging (also known as speckle statistics). Speckle statistics include acoustic structure quantification (ASQ) and Nakagami imaging; QUS spectroscopy; speed of sound (SoS); and shear wave elastography (SWE) metrics such as dispersion and viscosity. Emerging QUS techniques, integrating statistical methods most notably attenuation-based Nakagami imaging and backscatter-derived quantitative ultrasound spectroscopy show promise and could potentially become the noninvasive imaging method of choice in screening, grading, and monitoring NAFLD patients on therapy. These techniques compared to liver biopsy, could be implemented for screening purposes, compared to an ordinal scale, or provide an accurate continuous measurement of liver fat; the latter two would be most useful for the longitudinal follow-up of NAFLD patients to assess treatment response. Limitations of QUS techniques include confounding effects of body habitus and ascites. Further, QUS cannot simultaneously quantify fat in other organs as can be done with MRI based techniques. Finally, multiple simultaneously emerging QUS techniques from different vendors may prohibit widespread-buy in and may limit inter-vendor comparisons.

## Controlled attenuation parameter using transient elastography device

Controlled attenuation parameter (CAP) is the best-studied and clinically available technique for liver fat quantification, with the first clinical studies dating back to 2010 27. CAP is not a radiologic method, but rather a measure of ultrasound attenuation rate using a vibration-controlled TE device, commercially available as FibroScan™ (Echosens, Paris, France) 27. Transient elastography devices use different-sized probes that generate ultrasound pulses transmitted through tissue. The CAP is measured at the center frequency of the probe from the ultrasound data and correlates with the degree of ultrasound attenuation caused by intrahepatic fat accumulation. Results are reported in units of decibel per meter (dB/m), with values ranging from 100 to 400 dB/m, indicating lower to higher degrees of hepatic steatosis, respectively. Results of the diagnostic accuracy of CAP in the literature are generally positive but somewhat mixed. In a meta-analysis from 2014 (421 NAFLD patients out of 1771 total patients), CAP showed good sensitivity and specificity in distinguishing stage 1, 2 and 3 hepatic steatoses; however, this meta-analysis recommended against routine clinical use of this device because of high false-positive and false-negative rates 28. When validated against liver biopsy, CAP has been shown to have excellent diagnostic accuracy for detecting S1, S2, and S3 hepatic steatosis; AUCs range from 0.83 to 0.97, 0.70 to 0.97, and 0.61 to 0.82, respectively 29-37. Similarly, excellent results in detecting hepatic steatosis have been reported when CAP has been compared to MRI estimated proton density fat fraction (PDFF) 38,39, even in morbidly obese patients before and after bariatric surgery 40. In a recent prospective multicenter study of 393 adults with NAFLD, CAP showed moderate to high diagnostic accuracy in discerning S1, S2, and S3 hepatic steatosis.

Measuring CAP has multiple advantages, including simultaneous assessment of hepatic steatosis and fibrosis, which is valuable in predicting progression and treatment strategies in NAFLD. Measurement of CAP is typically performed by a hepatologist in an office setting in less than five minutes. Values are obtained from a 3 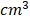 volume, which is significantly larger than the region analyzed through a liver biopsy, decreasing the potential risk for sampling bias. Limitations of CAP include poorly standardized cut-off values in classifying hepatic steatosis and the potential for CAP estimation of hepatic steatosis to be affected by differences in skin-to-capsule ratio, which may also confound other fat quantification methods. Furthermore, although CAP is an ultrasound technique, measurements are obtained without visualization of the liver. Thus, blind estimation of liver fat may result in inadvertent inclusion of masses, vessels, ducts, or uneven steatosis, any of which may limit accurate assessment. As such, there is a need for QUS parameters to be associated with radiological ultrasound machines to overcome these challenges.

## Attenuation and backscatter coefficient

**Figure 2:**
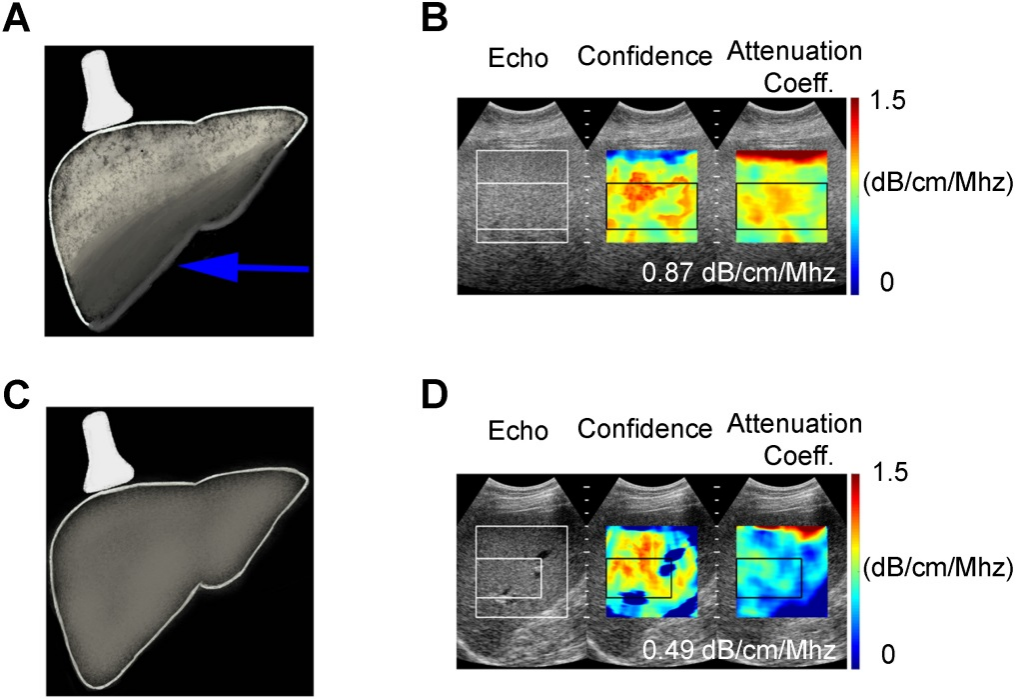
Attenuation Coefficient quantitative ultrasound method. Schematic **(A)** and clinical image **(B)** of a 55 year old female (BMI 43.5) with fatty liver demonstrating greater ultrasound beam attenuation within the deep aspects of the liver (arrow) and high attenuation coefficient of 0.87 dB/cm/MHz. Schematic **(C)** and clinical image **(D)** of 60 year old male (BMI 28.41) with normal liver demonstrating homogenous attenuation throughout the liver with a low attenuation coefficient of 0.49 dB/cm/MHz.

Acoustic waves are attenuated differently by steatotic versus normal liver parenchyma, and this difference is quantified in AC measurements. Unlike CAP, AC values are obtained from ultrasound systems (Figure 2). Attenuation coefficient has been well studied since the 1980s 41-44 and has been shown to correlate with the severity of hepatic steatosis 45-47. Recent studies have compared AC with MRI-PDFF and liver biopsy and reported excellent results 46,48. Even in patients with chronic liver disease (non-hepatitis B and C patients), diagnostic performance of AC has shown to be excellent in discerning grade ≥ 1, grade ≥ 2 and ≥ 3 steatosis when compared with MRI-PDFF 48. Although AC can reliably differentiate fatty liver, its accuracy is hampered by co-existing inflammation or fibrosis, which are often present in later stages of NAFLD. Consequently, considerable overlap in measurements can be seen with fatty and cirrhotic livers 49. Thus, other QUS parameters such as SWE should be included to quantify fibrosis and complement the role of AC in the diagnosis of NAFLD. Although ultrasound, in general, is affected by subcutaneous fat, AC measurements have been shown to have diagnostic value in obese patients 49. Attenuation imaging alongside shear wave elastography has been recently commercialized to assess the spectrum of liver diseases, including NAFLD and NASH 50. Attenuation coefficient measurements are obtained with clinically available ultrasound machines using grayscale images to guide measurements, thereby allowing the operator to select representative hepatic parenchyma and avoid masses, vessels, or ducts.

Backscatter coefficient (BSC) is another widely studied QUS parameter first described in 1973 51, in which backscattered signals from tissue are used to detect intra-hepatocyte fat. Fat vacuoles within hepatocytes increase ultrasound scattering signals, resulting in greater backscatter and a brighter (more echogenic) appearance of the liver. Backscatter coefficient has been studied in characterizing liver tissue architecture and quantifying liver fat with promising results 45,47,52-54, and more recently several studies have shown excellent diagnostic accuracy for BSC in detecting ≥ 5% hepatic steatosis compared to MRI-PDFF 46,55. Repeatability and reproducibility studies of AC and BSC techniques have similarly been tested and proven to be excellent with high interobserver and inter-platform reproducibility 56-58.

## Computerized hepatorenal index

**Figure 3:**
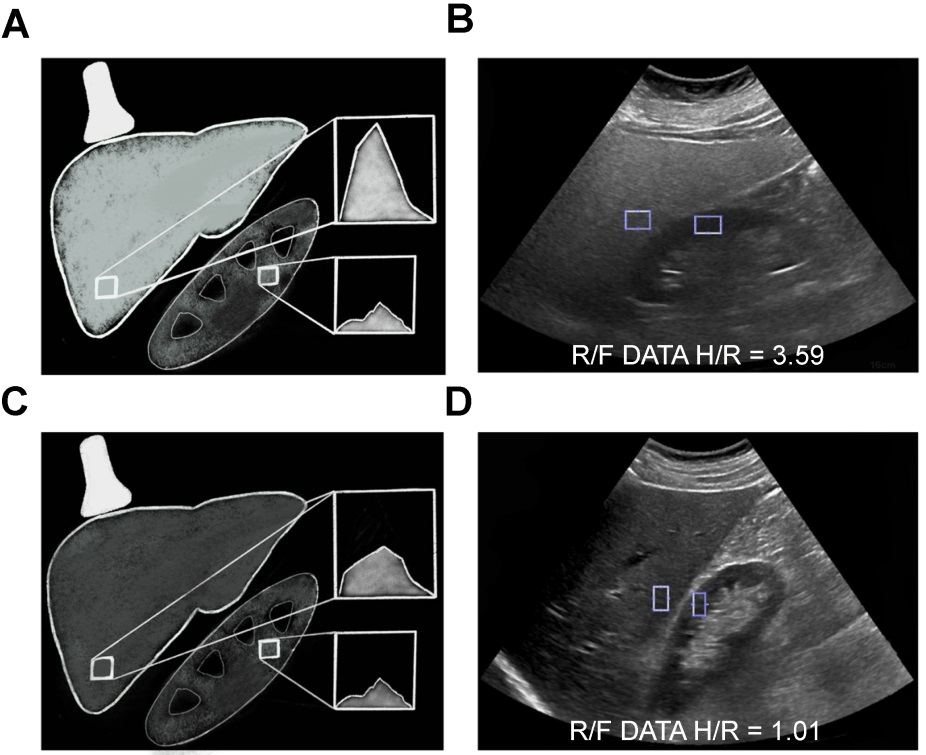
Computerized hepatorenal ratio quantitative ultrasound method. Schematic **(A)** and clinical image **(B)** of a 55 year old female (BMI 43.56) with fatty liver demonstrating increased echogenicity of the liver compared to the right kidney with the H/R ratio of 3.59. Schematic **(C)** and clinical image **(D)** of a 60 year old female (BMI 28.2) with normal liver demonstrating similar echogenicity of the liver compared to the right kidney with the H/R ratio of 1.01.

In conventional ultrasound, the diagnosis of hepatic steatosis is typically made by comparing the echogenicity of the liver and right kidney in the same image. Multiple factors affect this comparison, including variations in machine parameters such as gain/depth/power, time-gain- compensation settings and patient anatomy such as attenuation caused by rib shadow or subcutaneous fat, etc. These variations can result in variability of results 25. On the other hand, computerized calculation of the hepatorenal index (HRI) can provide a quantitative assessment of hepatic steatosis that corrects for potential variations in machine settings and body habitus. The HRI is calculated by drawing regions of interest in the liver parenchyma and right kidney at similar depths, and extrapolating ultrasound-beam parameters (Figure 3). Many previous investigators have manually placed ROI with this method because one can choose an area of the liver parenchyma that is void of blood vessels or lesions such as cysts or hemangiomas 59-61. The renal ROI is usually placed in the renal cortex between the pyramids at the same distance from the probe and near the center of the image to avoid distorting effects in ultrasonic wave patterns. From these ROIs, total brightness level, mean brightness level, standard deviation, most frequent brightness level, and histograms are computed. Computerized placement of the ROIs is not as popular in the literature but can be used to preliminarily place an ROI which can then be adjusted to avoid vessels or focal lesions. While the latter may streamline this quantification method, it adds an extra step in assessment.

Prior studies have shown an excellent correlation between HRI and fat fraction obtained through MRS 62 and liver biopsy even in mild hepatic steatosis, with AUCs as high as 99.2% 60 and 99.6% 59 in patients with or without a history of chronic liver disease, respectively. Moreover, a study evaluating this technique in a broad patient population that included patients with NAFLD, chronic hepatitis C, and hepatitis B found that HRI values are independent of obesity, inflammation, and fibrosis 60. Limitations of this method include poor performance in patients with chronic kidney disease; inability to compare the liver when the right kidney is absent or in an ectopic location or when there are intrinsic abnormalities in the liver or kidney parenchyma near the interface of the two organs; and the lack of standardized computer algorithms.

## Ultrasound envelope statistic parametric imaging

Ultrasound-based envelope statistical imaging, also known as speckle statistics, is based on the passive parametrization of ultrasonic speckle patterns using established statistical distributions/models, which in turn relate to the structural and acoustic properties of tissues (scatterer density and size) 63,64. Acoustic structure quantification (ASQ) is a relatively new commercially available quantification method (Aplio XG; Toshiba Medical Systems, Otawara, Japan) based on speckle statistics, which was first introduced and validated for assessing liver fibrosis 65,66. This method is based on the statistical analysis of the variance between theoretical and factual echo amplitude distribution 65,67,68. Preliminary animal studies have reported high diagnostic accuracy for ASQ in classifying hepatic fibrosis and in quantifying hepatic steatosis 67. A more recent study revealed a strong correlation between ASQ and CAP values in patients with < 25% hepatic steatosis 69, and showed moderate to high diagnostic accuracy for ASQ in detecting hepatic steatosis when compared to CAP as reference 69. When compared to MRS and HRI, ASQ has been shown to have high diagnostic accuracy (AUC of 0.96) in detecting >10% hepatic steatosis with significantly higher diagnostic accuracy than HRI (0.96 vs. 0.77, respectively) 68.

Nakagami imaging is one of the most popular model-based speckle statistic methods, first introduced in 2000 70. It is derived from the Nakagami distribution, which offers a somewhat generalized/universal distribution model, and is minimally affected by attenuation 71-73. Nakagami imaging has been studied to classify hepatic steatosis with promising results 74. Non-model-based speckle statistic methods such as Shannon entropy have also been studied for evaluating hepatic steatosis and found to have moderate diagnostic ability in suspected NAFLD patients 75. Unlike Nakagami, this method uses backscattered statistics to characterize tissue microstructure without considering data distribution. The overall advantage of statistical model-based techniques is that they are relatively unaffected by attenuation and are increasingly operator- independent. To date, only a few clinical studies have compared QUS envelope statistic techniques for liver fat quantification, mainly in Southeast Asia where the prevalence and etiology of NAFLD are different from Western countries and validation is not performed with biopsy or MRI/MRS estimated fat fraction. Further human studies are warranted to validate their potential widespread clinical use.

## Quantitative ultrasound spectroscopy

Quantitative ultrasound spectroscopy uses frequency-dependent parameters extracted from raw RF ultrasound data related to characterize tissue microstructures 76-79. A unique attribute of spectroscopy parameters is that they are normalized to tissue phantoms, which in turn reduces system-dependent variance, and renders the parameters independent of instrumental settings 63. Several spectral based parameters have been developed, all of which are either related to the underlying tissue scatterer size or concentration. The most common parameters include the spectral slope (SS), the 0-MHz spectral intercept (SI), the mid-band fit (MBF), which are related to the scatterer size (SI, SS), the acoustics scatterer concentration (MBF, SI), or differences in acoustic impedance between the scatterer and its surrounding medium (SI) . Other more advanced parameters with a theoretical framework relation to acoustic scatterers include the effective scatterer size (ESS) and effective scatterer concentration (ESC) 80-82**.** Only a few animal studies have explored the utility of spectroscopy techniques in evaluating fatty liver and demonstrated the potential of spectroscopy parameters to detect and characterize steatosis 83-85. More specifically, a recent animal study showed that SS and MBF parameters classified hepatic steatosis with an accuracy of 84% 83. Another study indicated that spectral-based parameters could detect fatty liver with 86% accuracy 84. Several major advances in spectroscopy based techniques such as the inclusion of machine learning algorithms have further improved this technique, and are currently being explored in the context of NAFLD tissue characterization 84. Quantitative ultrasound spectroscopy based parameters are new areas of research in evaluating hepatic steatosis, and clinical studies are warranted to investigate their potential use.

## Speed of sound

Speed of sound (SoS) measurement is a QUS parameter used for evaluating hepatic steatosis, and can be used to characterize tissue properties based on alterations in ultrasound echo wave speeds in various media 86,87. Speed of sound has been shown to decrease proportionally to increased liver fat content 88-91. A recent study of 17 patients has shown excellent results in differentiating hepatic steatosis for SoS compared to MRI-PDFF and biopsy (AUC of 0.942 and 0.952, respectively) 92. Consequently, further studies with a larger population of patients with suspected or known NAFLD is warranted to validate the clinical application of SoS. Limitations include potential confounding factors, such as inflammation, parenchymal edema, increase in intracapsular pressure, and changes in temperature, as higher temperatures increase SoS values and vice versa. While multiple studies in animals have used standardized temperatures to investigate SoS 91, accuracy could potentially be affected in quantifying hepatic steatosis in humans and needs further research.

## Elastography metrics such as elasticity, viscosity, and dispersion

**Figure 4:**
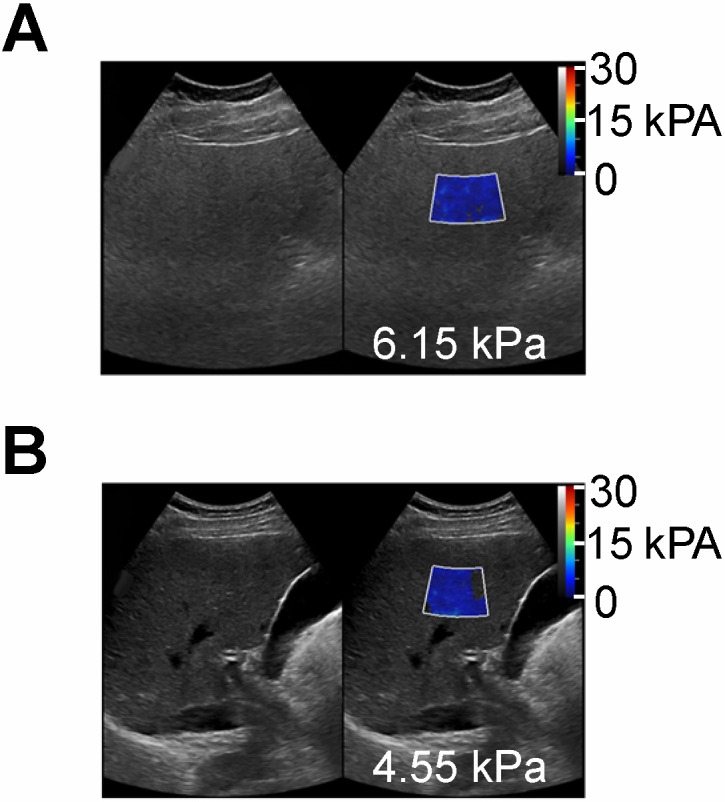
Shear wave elastography quantitative ultrasound method with calculated SWE measurements shown as color-coded scale superimposed on grayscale clinical images. **(A)** 55 year old female patient with NAFLD and MRI calculated fat fraction of 43 % with SWE measurement of 6.15 kPa and **(B)** a 60 year old male without history of NAFLD and MRI calculated fat fraction of 1.4 % with SWE measurement of 4.55 kPa. These SWE measurements show no significant differences (6.15 vs 4.55 kPa) despite marked variability in MR calculated fat fractions (43 % vs 1.4 %).”

Transient elastography and SWE are ultrasound-based elastography methods that measure liver stiffness, which have been successfully adopted clinically to detect and classify hepatic fibrosis 93,94. Transient elastography methods are the core technology in Fibroscan and have become clinical standard tools in hepatology to measure the liver stiffness in kPa 94,95. Shear wave elastography imaging (Figure 4) is an advancement of TE methods thought to be more quantitative than TE 94. Generally, one can measure the shear modulus from the traveling shear wave speed (generated mechanically in TE, or with an ultrasound pulse in SWE) by assuming the medium density (typically assumed to be 1000 kg/m3), mostly due to proportionality between shear wave speed and elastic modulus. Similar to TE, SWE is now commonly utilized in radiology clinics to classify fibrosis 94,96. Other related parameters such as shear wave viscosity and dispersion are being developed and suggested for measuring steatosis as well 97-99. A recent animal study investigated the viscoelasticity parameter, revealing significant changes in storage modulus of the livers in moderate-to-severe steatosis in comparison with normal livers; however, no significant differences were observed between different stages of steatosis 100. Another study compared shear wave velocity and dispersion slope to stage hepatic steatosis and fibrosis, using biopsy as the reference standard 101. The authors concluded that the use of elastic material models and group shear wave speed estimates appear to be sufficient for staging liver fibrosis (AUC of 0.9), while neither elastic nor viscoelastic shear wave-derived parameters correlated with hepatic steatosis in NAFLD patients (AUC of 0.5). Finally, a recent study compared ultrasound SWE-calculated fat fraction with MRS in 55 routine patients, and found no correlation between the two parameters 102. More recent investigations of hepatic viscosity in humans using shear wave propagation spectroscopy reveal moderate to high AUCs for shear wave viscosity in classifying hepatic fibrosis (0.64-0.87); however, this is a poor predictor for grading hepatic steatosis (AUCs under 0.64)^98^. Nonetheless, TE and SW measurements are highly promising in potential multiparametric approaches for simultaneous characterization of fibrosis and steatosis in NAFLD.

## Clinically Available Non-Ultrasound Based Imaging Methods for Hepatic Fat Quantification

### Magnetic Resonance Spectroscopy

**Figure 5:**
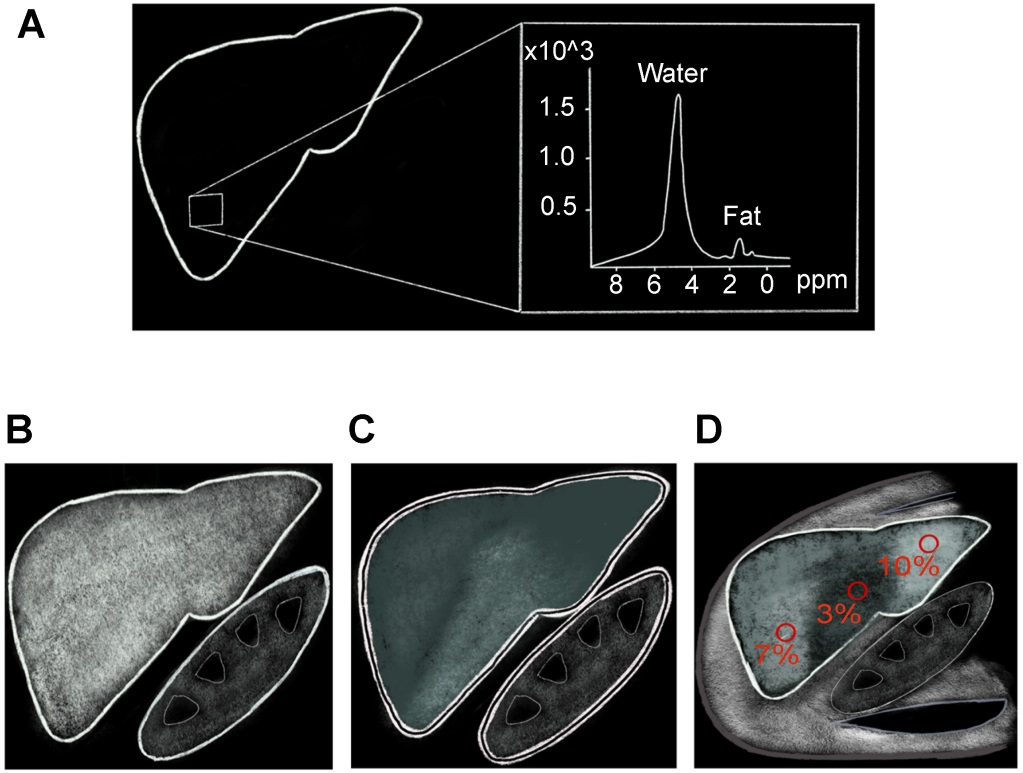
MR methods of hepatic fat assessment. **A)** MR spectroscopy calculates the hepatic fat fraction by separating out the number of water and fat protons in a small sample volume within the liver, which are demonstrated here as separate spectroscopy peaks. **B-D)** Chemical shift based MRI fat fraction, which is calculated by assessing signal loss on the Out-of-Phase **(C)** sequences when compared to In-Phase **(B)** sequences. **D)** Proton Density Fat Fraction percentage (PDFF) map is used to accurately calculate fat fraction by drawing ROIs on different areas of the liver as shown here.

Hepatic fat fraction estimated by MRS has proven to be an accurate substitute for liver biopsy and noninvasive imaging standard reference for liver fat quantification 103,104. This technique utilizes the chemical-shift phenomenon, where the nuclear MR spectral peak of fat is shifted in relation to water. Because most of the protons inside the liver parenchyma are contained in the water and fat, and signals from triglyceride can therefore be differentiated from water signal on MRS (Figure 5A). The sum of the fat proton signal intensities is divided by the sum of the fat and water protons signal intensities and is reported as hepatic fat fraction. Limitations of MRS include T1 and T2-relaxation effects, which can be overcome by using long repetition time/low flip angle and multi-echo MRS technique, respectively. In addition, MRS is limited by small sample volume, which could affect its accuracy in evaluation of patients with uneven fatty liver. Acquiring several MRS scans in different segments of the liver could potentially improve this limitation, but may be time-consuming to perform 105. Furthermore, this technique is limited to centers with MR spectroscopy expertise, which further limits its widespread clinical use.

### Magnetic Resonance Imaging

The MRI-PDFF is a newer method to estimate liver fat utilizing chemical-shift imaging (CSI) in a single breath-hold. Water protons precess faster than fat protons by about 3.5 parts per million (ppm) 106. Thus, signals originating from protons in fat and water can be added to create in-phase imaging (IP) or subtracted in out-of-phase imaging (OOP). Signal differences between IP (Figure 5B) and OOP (Figure 5C) images can then be used to calculate signal intensity from fat protons. Several studies have shown MRI-PDFF to have excellent correlation with liver biopsy 107,108. Potential biological confounders such as iron overload can be offset by simultaneously measuring and correcting for R2* dephasing effects 97. Magnetic resonance imaging estimated PDFF technique is superior to MRS and biopsy in assessment of uneven fatty liver disease, because multiple regions of interest can be analyzed simultaneously (Figure 5D). Disadvantages of MRI-PDFF include the fact that images are created from indirect calculations based on CSI measurements; thus, direct measurement of hepatic fat with MRS is still considered to be more accurate. Relatively high cost and limited availability, along with contraindications in patients with claustrophobia or implanted metallic devices, further limit the widespread use of MR-based imaging methods for screening or monitoring of NAFLD.

### Computed Tomography

Hepatic steatosis can be detected with unenhanced and contrast-enhanced computed tomography (CT) when absolute attenuation of the liver is ≤ 40 Hounsfield units (HU), especially when hepatic fat is > 30% 109. Hepatic steatosis can also be inferred when liver attenuation is at least 10 HU less than splenic attenuation 110. While CT is not typically performed as a primary modality to detect or diagnose hepatic steatosis, liver fat is often incidentally identified by CT performed for other reasons such as trauma, abdominal pain, or cancer staging. Unenhanced CT has proven to be more accurate than enhanced CT in evaluating hepatic steatosis 111, due to variations in contrast- enhancement kinetics of the liver and spleen. Additional variations in attenuation parameters are affected by high BMI, hepatic iron deposition, presence of underlying fibrosis or edema, and scanning parameters such as voltage, tube current, and pitch 112. Computed tomography HU measurements are based on tissue density, and small fractions of hepatic fat may have a negligible effect on attenuation, rendering it undetectable by CT 113. In addition, concerns of repeated exposure to ionizing radiation hinder the role of CT for continuous screening or monitoring of NAFLD. Variations in CT acquisition such as single source, dual-energy CT, and layered-detector CT have been utilized in hepatic fat detection, each with their own benefits and drawbacks. Detail of these CT technologies is beyond the scope of this paper but in brief, dual-energy CT either uses two separate energy sources or rapid kVp switching to obtain data. It relies on intrinsic differences in attenuation of materials at different X-ray spectra/tube currents 114, most notably iodine and water; however, this effect is less pronounced for water and fat. Newer techniques using multimaterial decomposition algorithms have been studied to calculate hepatic fat volume fraction and have yielded promising results compared to fat fraction derived from MRS and liver biopsy 115.

## Conclusion

In conclusion, nonalcoholic fatty liver disease is a major health issue with a worldwide increase in prevalence, paralleling the global obesity epidemic. Accurate noninvasive alternatives to liver biopsy in evaluating and monitoring levels of hepatic steatosis are have evolved significantly in the past decade. Emerging quantitative ultrasound-based approaches, integrating innovative statistical methods are promising new technologies to non-invasively assess hepatic steatosis.

## Figures and Tables

**Table 1:** Summary of major clinical studies on ultrasound-based liver fat quantification techniques

Parameters	Subjects	Diagnostic Performance (AUC, Sens, Spec, Correlation)	Reference Standard	Study
**CAP**	NAFLD (N=393)	AUC 0.76 for ≥ S1	Biopsy*	Siddiqui et al 2019 116
**CAP**	NAFLD (N=119)	AUC 0.80 and 0.87 for **≥ 5%** and **≥ 10%** steatosis, respectively	MRI-PDFF	Caussy et al 2018 38
**CAP**	NAFLD (N=76)	AUC 0.75, 0.74 and 0.82 for ≥ S1, ≥ S2, and S3 respectively (XL probe)	Biopsy*	Garg et al 2018 40
**CAP**	CLD and NAFLD (N=180)	AUC 0.84, 0.76 and 0.61 for ≥ S1, ≥ S2, and S3 respectively	Biopsy*	Chan et al 2018 35
**CAP**	NAFLD (N=55)	AUC 0.77, 0.78 and 0.78 for ≥ S1, ≥ S2, and S3 respectively	Biopsy*	Runge et al 2017 36
**CAP**	NAFLD (N=104)	AUC 0.85, 0.70 and 0.73 for ≥ S1, ≥ S2, and S3 respectively	Biopsy*	Park et al 2017 34
**CAP**	NAFLD (N=57)	AUC 0.94, 0.80 and 0.69 for ≥ S1, ≥ S2, and S3 respectively	Biopsy*	Chan et al 2017 33
**CAP**	NAFLD (N=261), multi-center	AUC 0.80 and 0.66 for ≥ S2, and S3 respectively	Biopsy*	de Lédinghen et al 2016 31
**CAP**	NAFLD (N=59)	AUC 0.83, 0.87, and 0.92 for **≥ 2%, ≥ 8%**, and **≥ 16%** steatosis, respectively	MRI-PDFF	Sasso et al 2016 39
**CAP**	NAFLD (N=183)	AUC 0.95, 0.85 and 0.72 for ≥ S1, ≥ S2, and S3 respectively	Biopsy*	Lee et al 2016 32
**CAP**	NAFLD (N=152)	AUC 0.88, 0.73 and 0.70 for ≥ S1, ≥ S2, and S3 respectively	Biopsy*	Imajo et al 2016 37
**AC/UGAP**	CLD (non-HBV, non-HCV) (N=126)	AUCs exceeding 0.87 (cutoff values for diagnosing steatosis grades ≥ 1, ≥ 2, and 3 were **5.2%**, **11.3%**, and **17.1%**, respectively)	MRI-PDFF	Tada et al 2018 48
**AC**	Healthy and CLD (N=65)	AUC 1.0	Biopsy*	Gaitini et al 2004 47
**AC and BSC**	NAFLD (N=61)	AUC - 0.78 (AC), AUC 0.85 (BSC) (MRI-PDFF cutoffs: **13.4%** and **16.8%**)	MRI-PDFF and Biopsy*	Paige et al 2017 46
**AC, BSC, and Texture**	NAFLD (N=80)	AUCs of 0.73 and 0.81 for mild and severe steatosis, respectively	US-FLI	Liao et al 2016 54
**BSC**	NAFLD (N=204)	AUC 0.98 for **≥5%** steatosis, Spearman ρ = 0.80; P < .0001, Sens-93% Spec-97%	MRI-PDFF	Lin et al 2015 55
**HRI**	Healthy and NAFLD (N= 127)	Sens 95.1%, Spec-100% for **≥ 9.15%** steatosis	MRS	Xia et al 2012 62
**HRI**	CLD and NAFLD (N=111)	AUC 0.992	Biopsy*	Webb et al 2009 60
**HRI**	Healthy and Diabetic (N=40)	AUC 0.996 for **≥5%** steatosis, Sens 100%, Spec 95%	MRS	Mancini et al 2009 59
**HRI**	Healthy (N=18)	Sens 66.7%, and Spec 100% for **≥5.56%** steatosis	MRS	Edens et al 2009 117
**Shannon entropy**	Healthy and Suspected NAFLD (N=394)	r = -0.630 (p < 0.0001)	US-FLI	Lin et al 2018 75
**ASQ**	CLD (N=89)	AUC 0.959 for **≥10%** steatosis, Sens 86.2%, Spec 100%, r = -0.87; P < .001)	MRS	Son et al 2016 68
**ASQ**	Healthy and Suspected NAFLD (N=67)	AUCs up to 0.8 for CAP values of < 250, 250 to 300, 300 to 350, and ≥ 350 dB/m Moderate correlation (*r* = 0.5) to MRS	CAP and MRS	Karlas et al 2015 69
**Nakagami**	Healthy (N=107)	r = 0.84 (p < 0.0001)	US-FLI	Wan et al 2015 74
**SoS**	Suspected NAFLD (N=17)	AUC of 0.952 v/s biopsy, and 0.942 v/s MRI-PDFF( **≥5%** steatosis)	Biopsy* and MRI	Imbault et al 2017 92
**SWE**	Healthy (N=55)	No correlation	MRS	Kramer et al 2017 102
**SWE**	NAFLD (N=135)	AUC 0.5, not significant	Biopsy*	Nightingale et al 2015 101
**SWE**	CLD (N=120)	No correlation	Biopsy*	Deffieux et al 2015 98

AC: attenuation coefficient; ASQ: acoustic structure quantification; AUC: area under the receiver operating characteristic curve; BSC: backscatter coefficient; CAP: controlled attenuation parameter; CLD: chronic liver disease; HRI; hepatorenal index; MRI: magnetic resonance imaging; MRS: magnetic resonance spectroscopy; NAFLD: nonalcoholic fatty liver disease; PDFF: proton density fat fraction; r: Pearson correlation coefficient; Sens: sensitivity; SoS: speed of sound; Spec: specificity; SWE: shear wave elastography; ρ: Spearman correlation coefficient; TE: transient elastography; UGAP: ultrasound-guided attenuation parameter; US-FLI: ultrasonographic fatty liver indicator. * ≥ S1, ≥ S2, and S3: fat accumulation in 5%-33%, 33%-66%, and >66% of hepatocytes, respectively, based on histologic analysis (ordinal scale). For non-biopsy gold standard references, cutoff values are listed in 3^rd^ column “diagnostic performance”.

## Abbreviations

AC: attenuation coefficient
ALT: alanine transaminase
ASQ: acoustic structure quantification
AUC: area under the receiver operating characteristic curve
BMI: body mass index
BSC: backscatter coefficient
CAP: controlled attenuation parameter
CLD: chronic liver disease
CSI: chemical-shift imaging
CT: computed tomography
ESC: effective scatterer concentration
ESS: effective scatterer size
HRI: hepatorenal index
HU: Hounsfield units
IP: in-phase
MBF: mid-band fit
MRI: magnetic resonance imaging
MRS: magnetic resonance spectroscopy
NAFLD: nonalcoholic fatty liver disease
NASH: nonalcoholic steatohepatitis
OOP: out-of-phase
PDFF: proton density fat fraction
QUS: quantitative ultrasound
RF: radiofrequency
SI: spectral intercept
SoS: speed of sound
SS: spectral slope
SWE: shear wave elastography
TE: transient elastography
US-FLI: ultrasonographic fatty liver indicator
